# Selection Bias, Vote Counting, and Money-Priming Effects: A Comment on Rohrer, Pashler, and Harris (2015) and Vohs (2015)

**DOI:** 10.1037/xge0000157

**Published:** 2016-05

**Authors:** Miguel A. Vadillo, Tom E. Hardwicke, David R. Shanks

**Affiliations:** 1 Primary Care and Public Health Sciences, King’s College London, London, United Kingdom; 2 Division of Psychology and Language Sciences, University College London, London, United Kingdom

**Keywords:** money priming, meta-analysis, *p*-hacking, publication bias, replications

## Abstract

When a series of studies fails to replicate a well-documented effect, researchers might be tempted to use a “vote counting” approach to decide whether the effect is reliable—that is, simply comparing the number of successful and unsuccessful replications. Vohs’s (2015) response to the absence of money priming effects reported by Rohrer, Pashler, and Harris (2015) provides an example of this approach. Unfortunately, vote counting is a poor strategy to assess the reliability of psychological findings because it neglects the impact of selection bias and questionable research practices. In the present comment, we show that a range of meta-analytic tools indicate irregularities in the money priming literature discussed by Rohrer et al. and Vohs, which all point to the conclusion that these effects are distorted by selection bias, reporting biases, or *p*-hacking. This could help to explain why money-priming effects have proven unreliable in a number of direct replication attempts in which biases have been minimized through preregistration or transparent reporting. Our major conclusion is that the simple proportion of significant findings is a poor guide to the reliability of research and that preregistered replications are an essential means to assess the reliability of money-priming effects.

Psychological science has been championing endeavors to examine the reliability of its most fundamental findings (Klein et al., 2014; Open Science Collaboration, 2015) and has been questioning the adequacy of mainstream research practices (Simmons, Nelson, & Simonsohn, 2011). The outcome of these efforts suggests that some popular textbook “facts” are either unreliable or radically inaccurate. Among the findings under dispute, priming effects (particularly so-called *social* priming effects, to distinguish them from *cognitive* priming effects, such as semantic or repetition priming) have become the center of an intense, and at times virulent, debate (Yong, 2012). Researchers working in this area have claimed that people’s judgments, decisions, and overt behavior can be dramatically swayed by exposure to subtle and seemingly irrelevant cues in the environment. For example, several studies have suggested that people are more generous after being primed with words related to religion (e.g., Shariff & Norenzayan, 2007). In recent years, research on priming has become increasingly influential, featuring in popular books (Thaler & Sunstein, 2008) and even in policy reports (Dolan, Hallsworth, Halpern, King, & Vlaev, 2010).

However, recent studies have suggested that this widespread enthusiasm for social priming may be unfounded. Numerous direct replication attempts have been unable to reproduce prominent priming effects (Doyen, Klein, Pichon, & Cleeremans, 2012; Gomes & McCullough, 2015; Klein et al., 2014; Pashler, Coburn, & Harris, 2012; Rohrer, Pashler, & Harris, 2015; Shanks et al., 2013, 2015), and there is growing concern that some are either substantially weaker than previously thought or are even entirely spurious. In the Many Labs project (Klein et al., 2014), for example, “flag priming” and “currency priming” effects could not be reproduced in the majority of direct replication attempts conducted by 36 independent laboratories.

Here, we focus on a particular episode of this debate that has taken place in the pages of this journal. In a series of four experiments (plus another two in the appendixes), Rohrer et al. (2015) reported that they could not reproduce a prominent money priming effect (Caruso, Vohs, Baxter, & Waytz, 2013). The archetypal observation in this literature is the apparent modification of participants’ behavior on a variety of measures following exposure to images of money, or tasks that involve subtle activation of the concept of money. For instance, it has been claimed that money priming causes people to become less willing to help others (Vohs, Mead, & Goode, 2006) and more likely to endorse the values of the free market economy (Caruso et al., 2013). Despite making extensive efforts to mimic the procedure of the original studies and to achieve high statistical power, none of the experiments conducted by Rohrer et al. yielded statistically significant results.

In response to this series of nonreplications, Vohs (2015) defended the reliability of money-priming effects by suggesting that the discrepancy in findings must be due to a number of moderators. For instance, participants in three of the five experiments reported by Caruso et al. (2013) may have been more inclined to have positive views about money because they were enrolled at the University of Chicago, an institution with a reputation for achievements in economics. Presumably, this could induce a greater susceptibility to money priming. Although speculative (see the Discussion section), postulating moderators is in principle defensible: If the original money-priming effects are reliable, then any failure to observe them must be due either to insufficient power or to some (known or unknown) moderators. This conclusion, however, is valid only if the reliability of the original findings is beyond dispute.

Vohs’s (2015) second line of argument is focused precisely on showing that the robustness of money priming is beyond dispute. Following a vote counting approach (Hedges & Olkin, 1980), Vohs provided a list of the effect sizes from 63 experimental contrasts that appear compellingly to confirm variations of the money-priming hypothesis. The core of this argument is that there are too many successful demonstrations of money priming to doubt their reliability. In her Table 1 (p. e88), she summarized studies in which the dependent variables were performance measures such as task persistence (usually increased by subtle money primes), and in Table 2 (pp. e89–e90) she reported interpersonal dependent measures such as helpfulness (usually decreased). Vohs concluded by noting the influence of money priming on a diverse set of dependent variables:

I did not have the space to cover the entirety of money priming experiments, which are 165 at last count. To name a few: Money priming mitigates the fear of death . . ., potentiates the persuasiveness of messages aimed at the self . . ., and curtails the savoring of experiences. . . . Money cues make people averse to others’ emotional expressiveness . . . and induce feelings of being physically colder than otherwise. . . . In the time I spent writing this commentary, multiple papers came across my desk relating money priming to trust, connectedness to the workplace after social ostracism . . ., and disinterest in religion. (p. e91).

[Table-anchor tbl1]

Such a large body of studies seems to provide powerful support for the reality of money priming. In the remainder of this comment, we ask whether the large number of “successful” demonstrations of money-priming effects confirms that these findings are indeed robust. Alternatively, is it possible that, despite their large number, previous reports of money priming mainly reflect false positives? There are no easy and unambiguous answers to these questions. However, the meta-analytic toolbox is well equipped to permit detection of biases that might artificially inflate the number of positive results.

In the following sections, we explore biases in different data sets of the money-priming literature using four meta-analytic methods. As we show, although these methods are based on different inputs (e.g., effect sizes, sample sizes, and *p* values) and make different assumptions, they all converge on a common conclusion: The evidence invoked by Vohs (2015) to support the robustness of money priming is compromised by selective reporting and other questionable research practices. In light of this evidence, we think that there are reasons to remain skeptical about the reliability of money priming.

## Funnel Plot Asymmetry of Money-Priming Experiments

Funnel plots provide a simple means to explore whether scientific findings reflect reliable effects or, alternatively, are undermined by publication or more generally selection biases (Egger, Smith, Schneider, & Minder, 1997) and/or by questionable research practices (Simmons et al., 2011). Other things being equal, one would expect to find more variable results among small and underpowered studies than among large studies: Experiments with large samples should yield very precise and reliable effect size estimates, whereas studies with small samples should yield less-precise and, consequently, more-variable estimates. If one plots the effect sizes of a set of experiments against their measurement precision, then one would expect to find a symmetric funnel-shaped distribution, with low variability across very precise studies and increasing variability as precision decreases. However, there is no a priori reason why the average effect size should vary across studies as a function of precision.

In contrast, if a set of studies is strongly biased by the decision to select only significant results, then their effect sizes will necessarily be related to measurement precision (Nosek, Spies, & Motyl, 2012; Rosenthal, 1979). The reason for this is that only very large effects can reach statistical significance in small (imprecise) studies, whereas even very small effects can be detected in large (precise) studies. Consequently, selection bias will induce a correlation between effect sizes and precision, so that smaller studies yield larger effects than do larger studies (Button et al., 2013). Although this correlation is typically considered an index of publication bias, it is an equally valid means to explore whether the experiments selected for inclusion in a narrative review, such as the one presented by Vohs (2015), which includes both published and unpublished research, represent a biased sample of the evidence.

**Figure 1 fig1:**
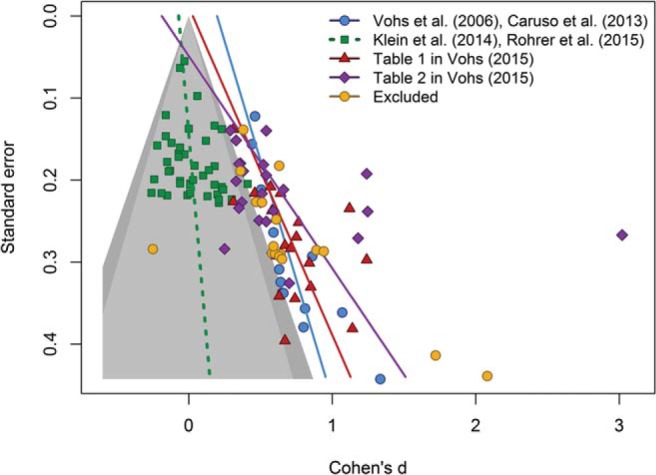
Contour-enhanced funnel plot of four data sets. The light gray area represents studies with *p* values larger than .10. The dark gray area represents marginally significant *p* values (i.e., .05 < *p* < .10). Lines represent Egger’s regression test for funnel plot asymmetry. See the online article for the color version of this figure.

In Figure 1 we plot the effect sizes of four data sets against the standard errors of the treatment effects (precision). The gray contour denotes the area in which effect sizes are nonsignificant in a two-tailed *t* test (α = .05**)**. The first set of effect sizes, represented by the darker circles (blue in the online version of the article), refers to the results of the seminal article on money priming (Vohs et al., 2006) and the original results of the study (Caruso et al., 2013) that Rohrer et al. (2015) attempted to replicate. As noted by Rohrer et al., visual inspection suggests that the effect sizes in this data set are strongly correlated with their standard errors. Egger’s regression test for funnel plot asymmetry (Egger et al., 1997) confirms that this relation is statistically significant, *t*(12) = 5.42, *p* < .001. The second set of effect sizes, denoted by squares (green online), comprises the effect sizes of the experiments reported by Rohrer et al., including not only the four experiments reported in their main text but also two additional experiments included in the appendixes. This set of effect sizes also includes 36 data points from the Many Labs project (Klein et al., 2014), which also attempted to replicate the results of Caruso et al. (2013). As can be seen, in this data set there is no evidence of asymmetry, *t*(40) = 1.48, *p* = .15, suggesting that these nonreplications are minimally affected by selection and reporting biases.[Fig-anchor fig1]

The two remaining data sets in Figure 1 refer to the effect sizes included in Vohs’s (2015) Tables 1 and 2.^1^ On the basis of the information available in those tables and in the articles where these effect sizes were originally reported, we coded all effect sizes as positive when they went in the direction predicted by the authors and as negative when they departed from the predictions. Several of the effect sizes included in Tables 1 and 2 were not statistically independent (i.e., they referred to different dependent variables collected on the same participants). To avoid giving undue weight to nonindependent effect sizes, we included in our analyses only the first effect size from each study. However, to confirm that this decision did not make an important difference to our conclusions, the excluded effect sizes are shown in Figure 1 as lighter circles (orange in the online article). Regression tests confirmed that the remaining effect sizes were also related to their standard errors, both for the studies included in Table 1 (triangles, which are red in the online article), *t*(18) = 3.87, *p* = .001, and those included in Table 2 (diamonds, which are purple in the online article), *t*(21) = 1.89, *p* = .072.^2^

**Figure 2 fig2:**
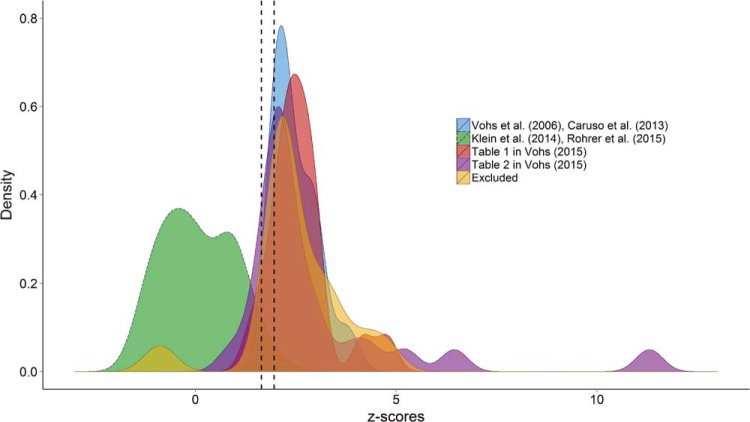
Kernel density plots of *z* scores in five data sets. The vertical dashed lines represent *z* scores of 1.64 and 1.96, respectively. All *z* scores to the right of the right line are statistically significant in a two-tailed test. The *z* scores between the lines are marginally significant in a two-tailed test. See the online article for the color version of this figure.

Beyond the quantitative results of Egger’s regressions, perhaps the most remarkable feature of Figure 1 is that many of the effect sizes that reached statistical significance are packed together immediately adjacent to the gray contour representing statistical significance. Funnel plot asymmetry is not a perfect indicator of selection and reporting biases (Ioannidis, 2005; Sterne et al., 2011), but the close alignment of effect sizes with the border of significance makes it difficult to believe that this distribution of effect sizes is unbiased. This is perhaps more clearly illustrated in Figure 2, which represents the density function of *z* scores within these data sets. We computed *z* scores by dividing each effect size by its standard error and fitted the density functions using a Gaussian kernel. As can be seen, the modal *z* scores in the studies included in Vohs’s (2015) Tables 1 and 2 are just large enough to be statistically significant in a two-tailed test. This is also the case for the small set of data points that we excluded from statistical analyses (because they were not independent from the others) and for the experiments reported by Vohs et al. (2006) and Caruso et al. (2013).[Fig-anchor fig2]

Overall, the patterns of data depicted in Figures 1 and 2 suggest that the effect sizes obtained experimentally by Vohs et al. (2006) and Caruso et al. (2013) and those listed by Vohs (2015) are likely to be biased either by the decision to select only significant results (Nosek et al., 2012; Rosenthal, 1979) and/or by questionable research practices such as selective reporting of significant outcomes, *p*-hacking (John, Loewenstein, & Prelec, 2012; Simmons et al., 2011), and hypothesizing after the results are known (HARKing; Kerr, 1998).

## Selection Models

Asymmetric funnel plots can arise for reasons other than selection and reporting biases (Ioannidis, 2005; Sterne et al., 2011). For instance, if researchers allocate more participants to experiments exploring small effects, then effect sizes and standard errors will be correlated even in the absence of selection bias (a compelling example can be found in the supplemental information of the Open Science Collaboration, 2015). In light of this shortcoming, it is always important to explore biases using alternative techniques that rely on different assumptions. Selection models provide another useful means to explore biases (Hedges, 1992; Sutton, Song, Gilbody, & Abrams, 2000). These models assume that the distribution of observed effect sizes depends not only on the average effect size of an area of research and its heterogeneity (as the random effects models used in meta-analyses typically do) but also on a weight function that determines how likely it is that a particular effect size will be selected for publication (or, in the present case, for discussion in a review), given its *p* value. Different selection models make different assumptions about the shape and properties of this weight function (Hedges & Vevea, 2005; Sutton et al., 2000), but in all cases, it is assumed that the best fitting parameters of the weight function can be used to estimate the potential impact of selection bias.

**Figure 3 fig3:**
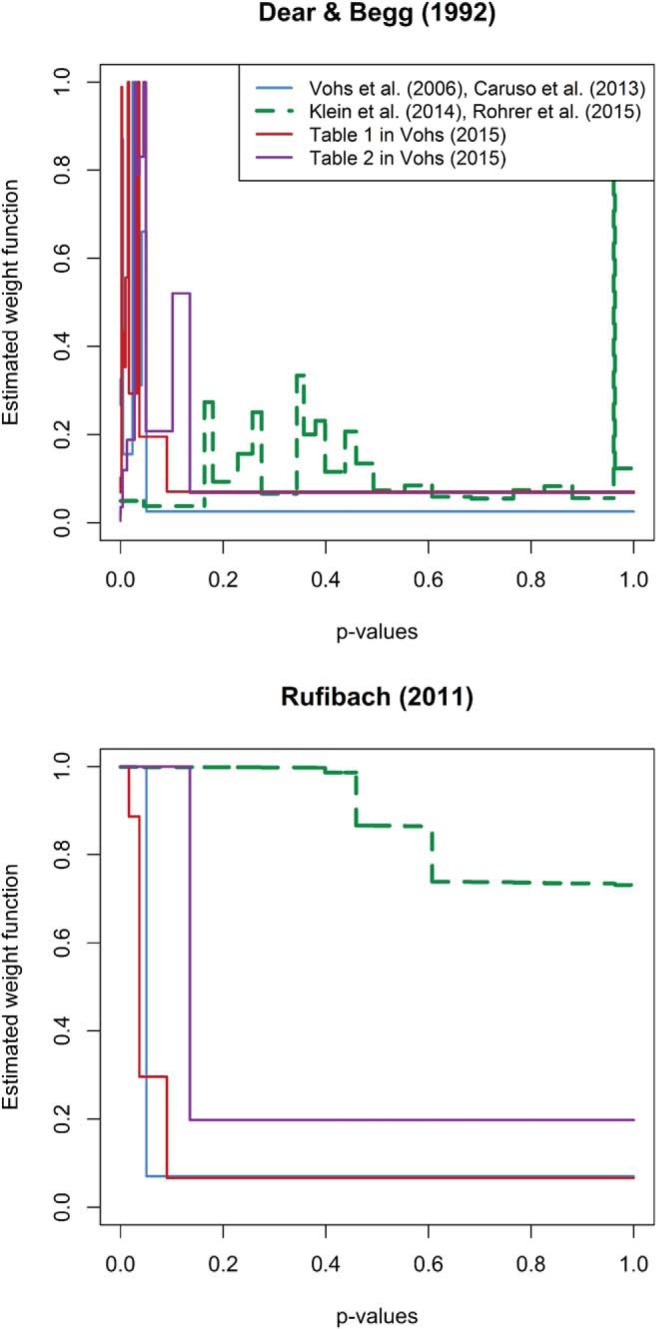
Best fitting weight functions of two selection models (Dear & Begg, 1992; Rufibach, 2011) applied to the four data sets shown in Figure 1. See the online article for the color version of this figure.

In Figure 3 we show the best fitting weight functions of two selection models (Dear & Begg, 1992; Rufibach, 2011) applied to the same four data sets that are included in Figure 1. These models were fitted using the maximum likelihood estimation procedure implemented in the selectMeta R package (Rufibach, 2011). The weight profiles provide an estimation of the likelihood that a result will be selected given the level of statistical significance it achieves. In the absence of selection bias, these profiles would be flat and the area below the weight function should be evenly distributed across all *p* values. In contrast, if the data are substantially affected by selection bias, small *p* values should be weighted more and, consequently, most of the area below the weight function should concentrate around small *p* values. As can be seen, both models suggest that *p* values play a crucial role in the distribution of effect sizes reported in Vohs et al. (2006) and Caruso et al. (2013), and in Tables 1 and 2 from Vohs (2015), but much less so in the Rohrer et al. (2015) and Klein et al. (2014) data. The best models of the former data sets are ones in which studies yielding *p* values greater than .1 are virtually guaranteed to be excluded. When the available data are so biased, it becomes difficult to estimate the true effect size.[Fig-anchor fig3]

It is important to note that the weight functions shown in Figure 3 do not simply reflect the fact that nonsignificant *p* values are abundant in Rohrer et al. (2015) and Klein et al. (2014) but rare in the Vohs (2015) data sets. Selection models do not yield a weight function with a sharp decline merely because there are many significant findings. When studies are high powered, selection models can fit the distribution of observed results without assuming an irregular weight function, even if most of them are statistically significant. Instead, selection models yield nonflat weight functions when the proportion of nonsignificant results is implausibly low given the observed distribution of effect sizes and sample sizes. Thus, the weight functions depicted in Figure 3 show that a distribution of results like the one presented by Vohs is unlikely to have arisen in the absence of selection bias.

## Test for Excess Significance

As an additional means to explore potential biases, we tested whether this set of studies contains an excess of significant findings. Psychological experiments are rarely adequately powered, where power is defined as the probability that the null hypothesis is rejected under a true experimental hypothesis. Average sample sizes are usually too small to warrant the conventionally prescribed level (.80) of statistical power (Button et al., 2013; Sedlmeier & Gigerenzer, 1989). This stands in stark contrast with the large proportion of published studies reporting significant results (see Fanelli, 2012). The discrepancy between studies’ average power and the observed number of significant results can be used as a proxy to estimate selection or reporting biases (Ioannidis & Trikalinos, 2007; Schimmack, 2012).

The first row in our Table 1 shows the proportion of significant results reported in the four sets of studies. The proportion of statistically significant findings in Vohs et al. (2006) and Caruso et al. (2013) is .86. However, the average observed power of the experiments reported in those articles is .68. Indeed, their average power to detect an effect of the size estimated with a random-effects or a fixed-effect meta-analysis is only .55. The proportion of significant results (.86) is thus larger than either of the power estimates. Even if the null hypothesis is false, it should not be rejected as frequently as it is in these studies, reflecting an “excess” of significant results above what would be expected, given the studies’ average power, and suggesting that the results of the experiments reported in those articles are “too good to be true” (Francis, 2012). The last two rows in Table 1 suggest that a similar trend is observed for the studies included in Vohs’s (2015) Tables 1 and 2 (see footnote 2), except for power estimates based on fixed-effect and random-effects effect size estimates in Table 2 (which are inflated by the inclusion of at least one clear outlier; see Figure 1).

## *p*-Curve Analysis

Recently, Simonsohn, Nelson, and Simmons (2014) have developed *p*-curve, a new tool to assess the evidential value of a series of statistical contrasts that is based on the distribution of *p* values. Unlike the tools used in the previous analyses, *p*-curve focuses only on statistically significant results and makes no assumptions about the distribution of nonsignificant results. If a series of significant statistical contrasts is exploring true effects, then very small *p* values (e.g., *p* < .025) should be more prevalent than are larger *p* values (e.g., .025 < *p* < .05). In contrast, if the real effect is zero and all significant results are false positives, then all *p* values are equally likely. The specific shape of the distribution of *p* values depends on the average statistical power of the contrasts included in the analyses: High-powered contrasts give rise to very steep right-skewed *p*-curves where most *p* values are very unlikely; underpowered experiments, on the contrary, give rise to flatter *p*-curves.

In employing *p*-curve, the statistical contrasts to be included in the analysis are not always the simple effects that researchers typically use to compute effect sizes. Therefore, conducting a direct *p*-curve analysis of the effect sizes listed in Tables 1 and 2 of Vohs (2015) would violate the core assumptions of *p*-curve. We therefore analyzed the original reports to extract the appropriate statistical contrasts. As explained in footnote 1, we were unable to access some of the unpublished studies included in Vohs’s Tables 1 and 2. Consequently, these could not be included in the analysis. Given that *p*-curve ignores nonsignificant results and that we were unable to access some unpublished experiments, we conducted a single analysis on all the available contrasts, without subdividing them into the categories used in Figures 1–3. Some of the experiments included in the *p*-curve analysis contained several statistical contrasts that were equally valid for our present purposes. When this was the case, we used the first contrast in the main analysis. To make sure that this decision did not influence the results, we conducted a robustness test in which these contrasts were replaced by the second valid contrast reported in those experiments. A *p*-curve disclosure table explaining how we selected each statistical contrast is available at https://osf.io/928r3/.

**Figure 4 fig4:**
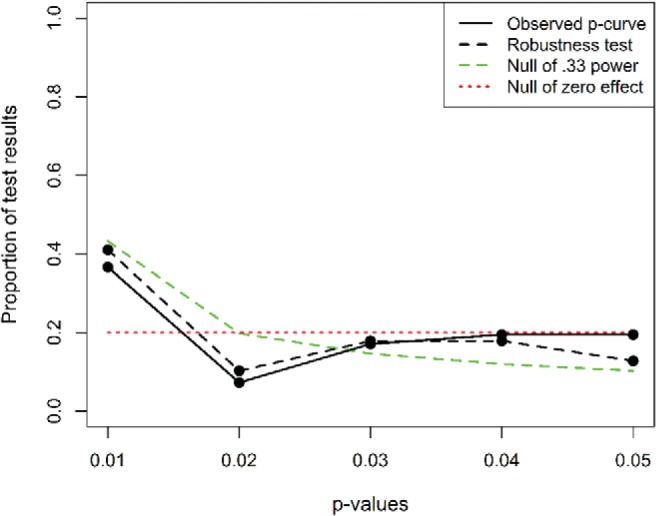
The *p*-curve of the key statistical contrasts for the studies included in Figure 1 whose main text was accessible. The *p*-curve disclosure table is available at https://osf.io/928r3/. See the online article for the color version of this figure.

Figure 4 shows the distribution of significant *p* values within this data set. As can be seen, both the main analysis and the robustness test suggest that the *p*-curve is rather flat, with only very small *p* values (*p* < .01) being slightly more prevalent. A continuous test using the Stouffer method suggests that the *p*-curve is significantly right-skewed for both the main analysis and the robustness test (*z* = −4.06, *p* < .0001, and *z* = −5.16, *p* < .0001, respectively). However, closer inspection of these results reveals that they are mainly determined by three contrasts (five in the robustness test) with very low *p* values. Given that *p*-curve is strongly biased by extreme *p* values (Simonsohn, Simmons, & Nelson, 2015), it is worth complementing these analyses with a simple nonparametric binomial test comparing the proportion of *p* values lower than .025 with the proportion of values between .025 and .050. This test yielded nonsignificant results for both the main analysis (*p* = .266) and the robustness test (*p* = .168).[Fig-anchor fig4]

Does this mean that these studies lack any “evidential value”? Not necessarily. Within null hypothesis significance testing, a null result is never evidence for the absence of an effect. As a practical means to test whether a set of studies lacks evidential value, Simonsohn et al. (2014) suggested testing whether the *p*-curve is even flatter than one would expect if studies were powered at .33 (i.e., if it is flatter than a very flat *p*-curve). A binomial test contrasting the observed distribution of *p* values against a null of .33 power was statistically significant (*p* = .032) for the main test and marginally significant (*p* = .076) for the robustness test, confirming this extreme flatness. In fact, the estimated average power of these studies, after correcting for selection bias, is only .18 for the main analysis and .25 for the robustness test. These power values, derived from the shapes of the observed *p*-curves, stand in stark contrast with the average observed powers that we computed in Table 1 without correcting for publication bias. This discrepancy is consistent with the hypothesis that the effect sizes of these studies are strongly biased.

Overall, these analyses yield mixed results. On the one hand, the significant results of the continuous test suggest that the studies included in this data set might be exploring real effects. However, this conclusion is heavily influenced by a small number of data points and is not supported by nonparametric binomial tests. Furthermore, the rather flat distribution of *p* values suggests that the evidential value is modest.^3^

## Discussion

It can be difficult to conceive that a scientific finding reported in dozens or even hundreds of experiments might not be reliable. Given just a few nonreplications and a long list of apparently successful studies such as the ones documented by Vohs (2015) in her Tables 1 and 2, it is tempting to conclude that money priming is a robust effect and that any failure to observe it must be due to a Type II error or to the presence of unknown moderators. However, meta-analysts have long known that this “vote counting” method is a poor approach for assessing the reliability of an effect (Hedges & Olkin, 1980). Simply contrasting the number of studies yielding significant versus nonsignificant results neglects the rich information conveyed by the distribution of effect sizes. For instance, a set of experiments can be dominated by null results even if a true effect does exist (Vadillo, Konstantinidis, & Shanks, 2016), and conversely, publication and reporting biases can give rise to a large number of significant findings in an area of research where real effects are very small or even completely absent (Shanks et al., 2015).

The analyses reported in the present article suggest that money-priming studies are likely to be influenced by selection bias, reporting biases, or *p*-hacking. Although the four methods we used (funnel plot asymmetry, selection modeling, testing for excess significance, and *p*-curve analysis) are based on different assumptions, they all converge in finding irregularity in the data. Metaregression methods ignore *p* values and evaluate the relationship between sample size and effect size in order to draw inferences about funnel plot asymmetry. Selection models use the effect sizes and their standard errors to draw inferences about the likelihood that studies yielding particular *p* values are selected. The test for excess significance uses sample size and effect size to determine whether the proportion of significant effects is greater than would be expected, given the experiments’ power. And *p*-curve ignores sample size and effect size to draw inferences about evidential value. In each case, evidence relevant to the existence of bias is the end result. These methods rest on different assumptions, and hence the conclusion cannot be attributed to any one particular, possibly disputable, set of assumptions. In the context of such reporting and selection biases, the sheer number of experiments yielding significant results and their average effect sizes provide little information about the reliability of a field of research.

It is doubtful whether known or unknown moderators should be invoked to explain the discrepancy in the results. If our confidence in money-priming effects were absolute, then any failure to observe it should necessarily mean that the replication was not properly conducted or that something important was different in the replication. However, as our confidence in the original finding decreases, the need to invoke moderators also declines. Furthermore, the potential moderators proposed by Vohs (2015) have not been reconciled with other evidence presented in favor of the money-priming hypothesis, nor has their influence been verified in any empirical enquiry. For instance, the assumption that money-priming effects might be strong only among students from institutions with a reputation in economics, such as the University of Chicago, might explain why the effect was absent in the participants tested by Rohrer et al. (2015). However, Vohs’s Tables 1 and 2 reveal that money-priming effects were seemingly found in very diverse populations, including not only university students from a wide range of institutions but also young children (Gasiorowska, Zaleskiewicz, & Wygrab, 2012). Speculation about potential moderators may be valuable as a means of hypothesis generation, but it is not an appropriate means of hypothesis confirmation (Kerr, 1998; Wagenmakers, Wetzels, Borsboom, van der Maas, & Kievit, 2012). If these extensions to the money-priming hypothesis are to have theoretical value, then they should (a) explain why other priming studies conducted in “a diverse range of locations . . . college students, working adults, children as young as 4 years old, and business managers” (Vohs, 2015, p. e87) were apparently not influenced by the proposed moderator and (b) generate new predictions that can be verified empirically (Lakatos, 1978; Meehl, 1990).

Of course, our analyses were not based on a systematic search of all the money-priming experiments, both published and unpublished, that have been conducted. Rather, we focused on the target studies conducted by Vohs et al. (2006) and Caruso et al. (2013) and in particular on those listed by Vohs (2015). An important goal for future research is to undertake a comprehensive formal meta-analysis, according to best practice guidelines (Lakens, Hilgard, & Staaks, in press; Moher, Liberati, Tetzlaff, Altman, & the PRISMA Group, 2009), including a clear description of the inclusion criteria and search procedure, as well as appropriate measures to explore biases and correct them. It is of course possible that such a comprehensive meta-analysis will yield conclusions different from those obtained here. For instance, an in-depth literature search might yield many unpublished studies (which typically observe smaller effect sizes than do published research; see Franco, Malhotra, & Simonovits, 2014; Polanin, Tanner-Smith, & Hennessy, in press) and/or additional published high-powered studies with large effect sizes. In either case, the consequence would be to attenuate or indeed even eliminate the funnel plot asymmetry shown in Figure 1. Whether such a meta-analysis would lead to a final comprehensive data set both lacking asymmetry and yielding a positive meta-analytic effect size is perhaps unlikely, but it would certainly extend the evidence base. Similarly, it would also be informative to confirm (or challenge) the results of our analyses with alternative tools for the exploration of selection and reporting biases (e.g., Copas, 1999; Guan & Vandekerckhove, 2016; van Assen, van Aert, & Wicherts, 2015).

In any case, our major conclusion is that the simple proportion or number of significant findings is a poor guide to the reliability of research. Nothing precludes the urgent need for more direct, preregistered replications of money-priming effects (van Elk, Matzke et al., 2015; Wagenmakers et al., 2012). Any narrative review of money priming that ignores the distortion induced by selection bias and questionable research practices is unlikely to paint a true picture of the evidence.

## Figures and Tables

**Table 1 tbl1:** Test of Excess Significance

Set of studies	Proportion of significant results	Mean observed power	Mean power to detect RE estimate	Mean power to detect FE estimate
Vohs et al. (2006); Caruso et al. (2013)	.86	.68, *p* = .122	.55, *p* = .017*	.55, *p* = .017*
Klein et al. (2014); Rohrer et al. (2015)	.02	.14, *p* = .998	.05, *p* = .884	.05, *p* = .884
Table 1 in Vohs (2015)	.85	.70, *p* = .103	.67, *p* = .060^†^	.65, *p* = .043*
Table 2 in Vohs (2015)	.79	.65, *p* = .096^†^	.84, *p* = .820	.77, *p* = .526
*Note*. Proportion of significant results in each data set and three measures of average power: (a) mean power to detect the effect size reported in each individual study, (b) mean power to detect an effect of the meta-analytic size estimated with a random-effects (RE) model, and (c) mean power to detect an effect of the meta-analytic size estimated with a fixed-effect (FE) model. The *p* values refer to the significance of one-tailed binomial tests contrasting the probability of the observed proportion of significant results given the three estimates of average power.				
^†^ *p* < .10. * *p* < .05.				

## Footnotes

1 Some of the studies included in Vohs’s (2015) tables were unpublished manuscripts or conference presentations. Although we were able to access most of them, we could not gain access to some reports. In one case (Mukherjee, Manjaly, Kumar, & Shah, 2015), we were unable to contact the authors of the study. In three cases (Park, Gasiorowska, & Vohs, 2015; Sarial-Abi, Hamilton, & Vohs, 2015; Sarial-Abi & Vohs, 2015), we were able to contact the first authors, but they preferred not to share their manuscripts or disclose more details of the statistical analyses. Although irrelevant for our present analyses, the authors alerted us that two of these papers (Sarial-Abi et al., 2015; Sarial-Abi & Vohs, 2015) actually refer to different experiments of a single manuscript in preparation. During the review of the present article, an anonymous reviewer revealed that the data reported by Chatterjee, Rose, and Sinha (2013) are under investigation because several lines of evidence suggest that they are tainted (Pashler, Rohrer, Abramson, Wolfson, & Harris, 2016). Consequently, in our analysis we omitted two data points from this article that were originally included in Vohs’s tables. Before conducting our analyses, we recomputed the effect sizes with the information provided in the original reports. In most cases, our estimations coincided exactly with the ones reported by Vohs or were reasonably close. However, in two cases, we observed important divergences, and consequently, we replaced the effect sizes reported by Vohs by our own estimations. A table with all the effect sizes included in the subsequent analyses is available at https://osf.io/9n2wh/.

2 Tests for publication bias tend to suffer from low statistical power. Therefore, it is common to adopt an alpha of .10 in these tests (Egger, Smith, Schneider, & Minder, 1997; Ioannidis & Trikalinos, 2007; Sterne, Gavaghan, & Egger, 2000). In the present article we present exact *p* values for all our tests of publication and reporting biases.

3 It is perhaps worth noting that statistical inference from *p*-curve can sometimes be unclear and misleading. The *p*-curve can be right-skewed even when the data are strongly biased by questionable research practices (Bishop & Thompson, 2015; Lakens, 2015). Furthermore the term *evidential value,* typically employed when a *p*-curve is significantly right-skewed, is ambiguous at best when used outside a purely Bayesian framework (E.-J. Wagenmakers, personal communication, January 21, 2016).
